# Interactions between Ciliate Species and *Aphanizomenon flos-aquae* Vary Depending on the Morphological Form and Biomass of the Diazotrophic Cyanobacterium

**DOI:** 10.3390/ijerph192215097

**Published:** 2022-11-16

**Authors:** Joanna Kosiba, Wojciech Krztoń, Judita Koreiviené, Sebastian Tarcz, Elżbieta Wilk-Woźniak

**Affiliations:** 1 Institute of Nature Conservation, Polish Academy of Sciences, Adama Mickiewicza 33, 31-120 Krakow, Poland; 2 Nature Research Centre, Akademijos Str. 2, LT-08412 Vilnius, Lithuania; 3 Institute of Systematics and Evolution of Animals, Polish Academy of Sciences, Sławkowska 17, 31-016 Krakow, Poland

**Keywords:** relationships, filamentous cyanobacteria, aggregated cyanobacteria, food web, protozooplankton

## Abstract

*Aphanizomenon flos-aquae* can form extensive blooms from freshwater to the brackish environment and, being a diazotrophic species, contribute significantly to the nitrogen and carbon cycle. It occurs as single filaments or aggregates and could be used as an alternative nutrients source for bacteria and ciliates. Ciliates are a group of organisms playing a crucial role in the transfer of nitrogen from primary producers to higher trophic levels in aquatic food webs. The aim of the experiment was to study the effects of the cyanobacterium *A. flos-aquae* on the community of five ciliate species (*Spirostomum minus*, *Euplotes aediculatus*, *Strobilidium* sp., *Vorticella* sp. and *Paramecium tetraurelia*). The response of each species to the presence of a low/high cyanobacterial biomass and to the different morphological forms of *A. flos-aquae* (single filaments or aggregates) was demonstrated. The results of the experiment showed the variability of interactions between the cyanobacterium *A. flos-aquae* and ciliates and pointed out the possible benefits that *A. flos-aquae* provides to the ciliates (e.g., a substrate for the development of bacteria as food for ciliates or as a source of nitrogen and carbon).

## 1. Introduction

Cyanobacteria are photosynthetic prokaryotic organisms that appeared on Earth about 3.5 billion years ago [1]. They are morphologically diverse and include unicellular and colonial to multicellular filamentous species [2]. Some of the cyanobacterial species (e.g., *Aphanizomenon*, *Anabaena*, *Cylindrospermopsis* and *Dolichospermum*) are diazotrophic and they are among the most important groups fixing molecular nitrogen in freshwaters [3]. Biological nitrogen fixation (conversion of dissolved N_2_ gas to ammonia by microorganisms) is an important process in global biogeochemical cycles that significantly offsets nitrogen losses through denitrification and an anaerobic ammonium oxidation [4]. Nitrogen fixed by cyanobacteria is released to water in the form of bioavailable ammonia and dissolved organic nitrogen that boosts the primary and secondary production [5]. The nitrogen pool is a potentially important source of nitrogen for other organisms in the pelagic food web [6]. Diazotrophically fixed nitrogen can be transferred to mesozooplankton directly by grazing on fresh or decaying nitrogen-fixing cyanobacteria [7], or indirectly via trophic vectors such as diatoms and ciliates [8]. Agglomerations of *Aphanizomenon flos-aquae* Ralfs ex Bornet & Flahault 1886 are unavailable to mesozooplankton due to their morphology (they are difficult to ingest). Therefore, microbial loop components, including ciliates, start to play a crucial role when *A. flos-aquae* forms blooms.

Ciliates are an important link to predatory copepods in eutrophic aquatic ecosystems as well as a significant pathway of energy flow [9]. They are an essential link to mesozooplankton, transferring up to 90% of fixed N_2_ via the microbial loop (see references, e.g., in [10]). The microbial loop was not discovered until the 1980s [11,12]. At that time, it was also recognized that ciliates were an important component of the aquatic food web, being important consumers of bacterioplankton and transmitters of energy from bacteria and phytoplankton to higher level consumers [13]. However, only in 2012 were ciliates included in the PEG model [14]. Despite the fact that ciliates are structural elements of the aquatic food web and are essential for the cycling of matter in all types of aquatic ecosystems, they still receive little attention in ecological studies [15].

Not much attention has been paid to the relationships between diazotrophic cyanobacteria and ciliates, although ciliates play a crucial role in transferring nitrogen to higher trophic levels. On the other hand, there are no studies that address the possible advantages provided by cyanobacteria to ciliates, such as food providing, etc.

Since one of the diazotrophic cyanobacteria, *Aphanizomenon flos-aquae*, known from temperate [16] and high latitudes [17] has shown negative effects on ciliates [18], we would like to continue the research on the interactions between *A. flos-aquae* and ciliates. *A. flos-aquae* forms blooms in brackish waters, e.g., of the Baltic Sea [19], and in freshwaters [16,20]. The filamentous cyanobacterium can occur as single filaments or form aggregates that enable species to float and form biomass scums on the water surface. The ability to photosynthesize and fix atmospheric nitrogen makes this species a source of nutrients, e.g., nitrogen and carbon. In fact, Christoffersen et al. [13] observed large fluxes of carbon between bacteria, ciliates and crustaceans during and after an *Aphanizomenon* bloom.

In our study, we assessed if there is a specific interaction between ciliates and *A. flos-aquae* with a ‘high’ and ‘low’ biomass and different morphological forms (single filaments or aggregates). The existing literature is not consistent on this issue. The mesocosm experiment of Engström-Öst et al. [21] showed a higher abundance of bacteria and a higher density of ciliates after the decay of *A. flos-aquae*. On the other hand, Sellner [22] observed that the abundance and activity of bacteria and protists were lower when *A. flos-aquae* aggregates dominated. Therefore, we hypothesized that a high biomass of single filaments of *A. flos-aquae* promotes an increase in the density of the ciliate groups, while a high biomass of the aggregated filaments of *A. flos-aquae* inhibits the development of ciliates.

The result of the study will improve the understanding of aquatic ecosystems from the perspective of the heterogeneity of thin and small patches of the aquatic microenvironment: the organic matter, nutrients and microorganisms and the effects of *A. flos-aquae* on ciliates.

## 2. Material and Methods

The experiment was performed with four strains of *Aphanizomenon flos-aquae* and a freshly collected sample containing five species of ciliates, which was maintained as a natural culture (enriched with dried hay) for two weeks. Two strains of *A. flos-aquae* were isolated from an artificial pond (Podkamycze 1) (southern part of Poland; 50°05′11″ N, 19°50′01.6″ E) (strain of single filaments of *A. flos-aquae* 2016/PL/F5 and strain of aggregated to bundles *A. flos-aquae* PL 2016/PL/E3) and two strains from the Curonian Lagoon (Lithuania, N 55°30′, E 21°15′) (strain of single filaments of *A. flos-aquae* 2012/KM/D3 and strain of aggregated to bundles *A. flos-aquae* 2015/KM/E8). The strains were isolated from the fresh samples using a glass microcapillary pipette, inoculated into an AF6 modified medium. The *A. flos-aquae* cultures were not axenic, however, they were re-isolated before the experiment to obtain a low bacteria density in the culture. The biomass of each *A. flos-aqua* culture was evaluated under a light microscope at 200× magnification. The density was counted in a Nageotte chamber (volume 0.05 cm^3^), estimating over 300 filaments per sample. The length and width of at least 25 filaments were measured for the biomass calculation. The biomass was calculated from the number of filaments counted and the average biovolume of the filaments estimated using the geometric shape formula [23,24].

The ciliate natural culture consisted of five species: *Euplotes aediculatus* Pierson, 1943 + *Spirostomum minus* Roux, 1901+ *Strobilidium* sp. + *Paramecium tetraurelia* Sonneborn 1975 + *Vorticella* sp. The species were cultured, as a whole community, in the Institute of Nature Conservation, Polish Academy of Sciences. The ciliates were identified according to the taxonomic keys [25,26] and two of them (*S. minus* and *P. tetraurelia*) were subjected by a molecular analysis (description below). The ciliates represented different species. They were: 1. *Spirostomum minus*: bacterivorous, fast swimming, a large cell size of 400 to 600 μm, a very elongated body and it lives in fresh and marine waters [25,26]; 2. *Euplotes aediculatus*: omnivorous, free swimming, a cell size of 105 to 165 μm, the cells are dorsoventrally flattened with a very large mouth area (peristome) and the species are widely distributed in marine and freshwater environments [25,26]; 3. *Strobilidium* sp.: mixed feeding on algae and bacteria, very fast swimming, rotates in one position in the water, very small cells of 35 to 60 μm, somatic cilia spirals around the body [25,26] and they are distributed in marine and freshwater environments; 4. *Vorticella* sp.: bacterivorous, lives in water bodies such as ponds, lakes, also in marine water as well as in aquatic vegetation, they are sessile ciliates, usually attached to substrates through their stalk and the cells are 40–80 μm in size [25,27]; and 5. *Paramecium tetraurelia*: bacterivorous, free-living in freshwater environments, a cosmopolitan species which can be easily grown in the laboratory and the cells are 100–180 μm in size [25,28]. At the beginning of the experiment, the ciliates were cultured with a bit of dried hay.

Ciliates and cyanobacteria were conditioned before the experiment. Cyanobacteria were maintained in modified AF6 medium at 20 °C, illuminated with 40 μmol photons m^−2^ s^−1^ and with a photoperiod regime of 12/12 h light/dark cycle. Ciliates grew in a Żywiec brand mineral water at room temperature and 12/12 h photoperiod under light/dark conditions in the growth chamber (Fitotron SGC 120 Loughborough Technology Centre, UK). 1 mL of water from the culture containing the similar number of ciliates was added to each well of the plates. The experiment was performed in sterile 6-well plates for TC adherent culture, flat bottom, growth area 9.5 cm^2^.

The experiment was set up as follows:

1. The control sample: no cyanobacteria + 1 mL of community of ciliates + 9 mL of Żywiec brand mineral water (composition in 1 L of water: 281.00 mg/L of bicarbonate anion; 29.50 mg/L of magnesium cation; 59.30 mg/L of calcium cation; 1.70 mg/L of sodium cation; and 408.20 mg/L of total minerals).

Further samples consisted of single filamentous or aggregated *Aphanizomenon flos-aquae:*

2a. One mL of community of ciliates + 9 mL of medium (Żywiec brand mineral water) + 1 mL of culture of filamentous *A. flos-aquae* from strain PL (F5) (‘low’ biomass),
2b. One mL of community of ciliates + 9 mL of medium (Żywiec brand mineral water) + 1 mL of culture of filamentous *A. flos-aquae* from strain PL (F5) (‘high’ biomass),
3a. One mL of community of ciliates + 9 mL of medium (Żywiec brand mineral water) + 1 mL of culture of filamentous *A. flos-aquae* from strain LT (D3) (‘low’ biomass),
3b. One mL of community of ciliates + 9 mL of medium (Żywiec brand mineral water) + 1 mL of culture of filamentous *A. flos-aquae* from strain LT (D3) (‘high’ biomass),
4a. One mL of community of ciliates + 9 mL of medium (Żywiec brand mineral water) + 1 mL of culture of aggregates *A. flos-aquae* from strain PL (E3) (‘low’ biomass),
4b. One mL of community of ciliates + 9 mL of medium (Żywiec brand mineral water) + 1 mL of culture of aggregates *A. flos-aquae* from strain PL (E3) (‘high’ biomass),
5a. One mL of community of ciliates + 9 mL of medium (Żywiec brand mineral water) + 1 mL of culture of aggregates *A. flos-aquae* from strain LT (E8) (‘low’ biomass),
5b. One mL of community of ciliates + 9 mL of medium (Żywiec brand mineral water) + 1 mL of culture of aggregates *A. flos-aquae* from strain LT (E8) (‘high’ biomass).

The biomass of *A. flos-aquae* was used as follows. For the ‘high’ biomass experiment, 10 mL of the culture was taken (an average culture biomass of 2.59 ± 1.41 mg/mL). Then, 3 mL of the cultures with a ‘high’ biomass of *A. flos-aquae* were diluted 10 times with Żywiec water to obtain a ‘low’ cyanobacterial biomass for the experiment. At the beginning of the experiment, each prepared strain sample with a ‘low’ and ‘high’ biomass was shaken to obtain a homogeneous sample, and 1 mL was used for each treatment. Three replicates of each variant were performed. The main objective of the current study was to evaluate the different types of the agglomeration and biomass concentration on the ciliates, therefore, the *A. flos-aquae* biomass were evaluated only at the beginning of the experiment. All four strains were cultured, and the experiments were performed separately. However, since there were no differences in the ciliates’ response, we finally used the data from the unified filamentous (PL + LT *A. flos-aquae*) and aggregated (PL + LT *A. flos-aquae*) samples for further statistical analyses.

For ciliates’ counting, 1 mL of the sample was taken from every replicate (3 replicates were counted for each type of treatment). Each replicate was counted separately. An average value was then calculated. Each replicate was fixed with Lugol’s and counted in a 1 mL Sedgewick–Rafter chamber under a coverslip using a Nikon eclipse Ti-S light microscope.

The experiment lasted for two weeks. The density of the ciliates was counted every 3 days (period sufficient for the ciliate’s development). The density of the ciliates is shown in the Supplementary Materials.

## 3. Molecular Data Analysis

The genomic DNA was isolated using a Genomic Micro AX Tissue Gravity (A&A Biotechnology, Gdańsk, Poland) following the manufacturer’s instructions for the DNA isolation from the cell cultures. Both the quantity and purity of the extracted DNA were evaluated with a NanoDrop-2000 Spectrophotometer (Thermo Scientific, Waltham, MA, USA). The SSU rDNA fragment containing hypervariable domain V4 was amplified with forward TAReuk454FWD1 universal eukaryotic primers: a forward (5′-CCAGCA(G/C)C(C/T)GCGG-TAATTCC-3′) and a reverse TAReukREV3 primer (5′- ACTTTCGTTCTTGAT(C⁄T)(A⁄G)A-3′, using the protocol previously described by [23]. The PCR amplification was carried out in a final volume of 40 μL containing 30 ng of DNA, 1.5 U Taq-Polymerase (EURx, Gdańsk, Poland), 0.8 μL of 20 μM of each primer, a 10 Χ PCR buffer and 0.8 μL of 10 mM dNTPs. In order to assess the quality of the amplification, the PCR products were electrophoresed in 1% agarose gel for 30 min at 85 V with a DNA molecular weight marker (Mass Ruler Low Range DNA Ladder, Thermo Fisher Scientific, Waltham, MA, USA). For purifying the PCR products, 5 µL of each were mixed with 2 µL of Exo-BAP Mix (EURx, Gdańsk, Poland), and then incubated at 37 °C for 15 min and afterwards at 80 °C for another 15 min. The cycle sequencing was done in both directions with BigDye Terminator v3.1 chemistry (Applied Biosystems, Waltham, MA, USA). The primers used in the PCR reactions were again used for the sequencing. The sequencing reaction was carried out in a final volume of 10 μL containing 3 μL of template, 1 μL of BigDye (1/4 of the standard reaction), 1 μL of sequencing buffer, and 1 μL of 5 μM primer. The sequencing products were precipitated using an Ex Terminator (A&A Biotechnology, Gdańsk, Poland) and separated on an ABI PRISM 377 DNA Sequencer (Applied Biosystems, USA).

The sequences were examined using Chromas Lite (Technelysium, South Brisbane, Australia) to evaluate and correct the chromatograms. The alignments of the studied sequences were performed using BioEdit software version 7.2.5 [29] and checked manually. All the obtained sequences were unambiguous and were used for the analyses. The species identification was done with BLAST (Basic Local Alignment Search Tool, an on-line tool of National Library of Medicine, Bethesda, MD, USA) [30].

The sequences are available from the NCBI GenBank database (accession numbers:

*Paramecium tetraurelia*https://www.ncbi.nlm.nih.gov/nuccore/OP727309.1/, accessed on 31 October 2022;

*Spirostomum minus* https://www.ncbi.nlm.nih.gov/nuccore/OP727310.1/, accessed on 31 October 2022).

## 4. Statistical Analysis

We used a generalized linear model (GLM) to test the relationship between the ciliate natural culture and the biomass of the cyanobacterium *A. flos-aquae*. GLM is an extension of the simple linear regression model for a continuous response variable given with one or more continuous and/or categorical predictors. We calculated the GLM using the Poisson distribution and the dependent variables were the *A. flos-aquae* total biomass and *A. flos-aquae* morphology and its effect on the particular ciliate species. We checked also the effect of a ‘high’ or ‘low’ biomass of *A. flos-aquae* on the density of the ciliate species and the effect of the morphology of the *A. flos-aquae* (single filaments/ aggregates) on the ciliate species.

The plots of the predicted values were generated using the package ‘ggeffects’. Data were considered statistically significant at *p* < 0.05. R-Studio, R v. 4.0.2 [31] was used for the statistical analyses.

## 5. Results

The results of the experiment showed that the total cyanobacterial biomass (*A. flos-aquae*) significantly affected four out five of the ciliate species: *Spirostomum minus*, *Euplotes aediculatus*, *Strobilidium* sp. and *Vorticella* sp., but not *Paramecium tetraurelia* (Table 1). However, the morphology of the filaments (single filaments/aggregates in bundles) significantly affected *Spirostomum minus*, *Euplotes aediculatus* and *Strobilidium* sp., but not *Vorticella* sp. nor *Paramecium tetraurelia* (Table 1).

A further statistical analysis (GLM) showed a positive significant effect of the ‘high’ biomass of *A. flos-aquae* on the density of the two species: *S. minus* and *E. eadiculatus*. The ‘low’ biomass of *A. flos-aquae* had a negative significant effect on the density of the *Strobilidium* sp. and a positive significant effect on two ciliate species: *S. minus* and *Vorticella* sp. The biomass of the cyanobacteria had no significant effect on the density of the *P. tetraurelia* (Table 2).

The statistical analysis (GLM) also showed a positive significant effect of the aggregated *A. flos-aquae* on *S. minus* and *Vorticella* sp., and a negative significant effect on the density of the *Strobilidium* sp. However, the biomass of the single filaments of *A. flos-aquae* had a positive significant effect on the density of the two ciliate species: *S. minus* and *E. aediculatus*. Neither the single filaments nor the aggregated filaments of *A. flos-aquae* had a significant effect on the density of the *P. tetraurelia* (Table 3).

**Figure 1:**
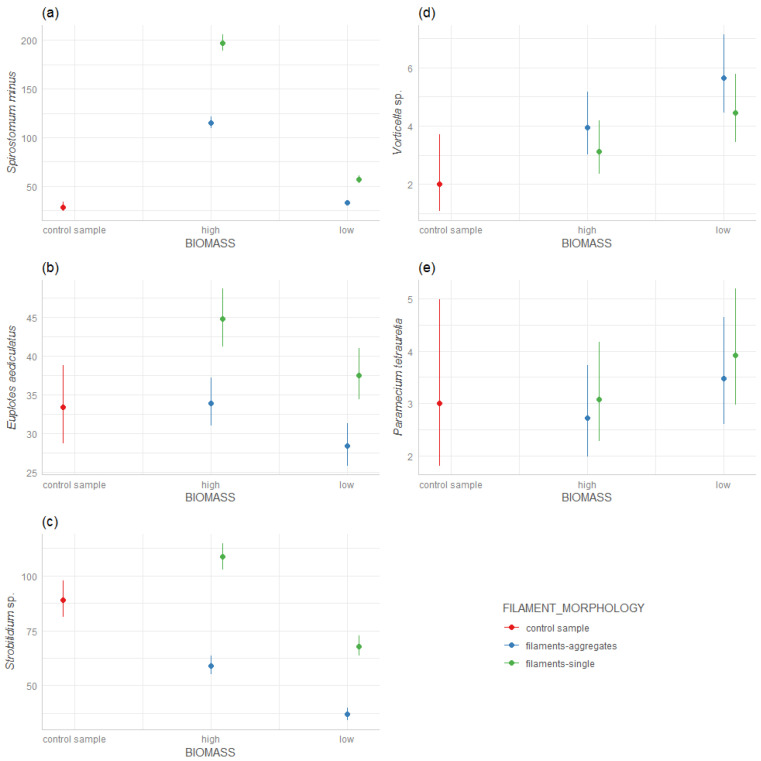
Response of ciliate species density on *A. flos-aquae* biomass (‘high’/‘low’) and filaments morphology (single filaments/aggregates): (**a**) *Spirostomum minus*, (**b**) *Euplotes aediculatus*, (**c**) *Strobilidium* sp., (**d**) *Vorticella* sp., (**e**) *Paramecium tetraurelia*.

We found that *Spirostomum minus* preferred a ‘high’ biomass of the filamentous *A. flos-aquae* strains (Figure 1a), whereas the ‘high’ biomass of the *A. flos-aquae* aggregates indicated poorer conditions for the development of *S. minus*. When the biomass was low for both filamentous and aggregated *A. flos-aquae*, *S. minus* developed only in a low abundance.

*Euplotes aediculatus* also developed well in the presence of the ‘high’ biomass of filaments strains *A. flos-aquae* (Figure 1b), and the ‘low’ biomass of the filamentous *A. flos-aquae* showed worse conditions for the development of *E. aediculatus*. It did not prefer developing in the presence of aggregates, both of a ‘low’ and ‘high’ biomass (Figure 1b).

*Strobilidium* sp. also developed well with the ‘high’ biomass of the filamentous *A. flos-aquae*. However, the ‘low’ biomass of the single filamentous and the ‘high’ and ‘low’ biomasses of the aggregates were the worse conditions even compared to the control probe (Figure 1c).

*Vorticella* sp. showed a different trend compared to *Spirostomum*, *Strobilidium* and *Euplotes*. The highest development of *Vorticella* was found in the ‘low’ biomass of aggregates *A. flos-aquae*, while the ‘high’ biomass of the aggregates and the ‘high’ and ‘low’ filamentous *A. flos-aquae* were less preferred by *Vorticella* sp. but they still formed in significantly better conditions than in the control probe (Figure 1d).

*Paramecium tetraurelia* showed a similar trend as *Vorticella* sp.—the highest density of *P. tetraurelia*—was found at a ‘low’ biomass of *A. flos-aquae* as the aggregates. However, compared to the control sample, the differences between the ‘high’ and ‘low’ biomass of *A. flos-aquae* and the type of filament morphology were not significant (Figure 1e) and since SD was high in the control sample, the interpretation is difficult.

## 6. Discussion

Cyanobacterial blooms on the surface of water form highly productive patches of different physical and chemical microenvironments [32]. These diverse microenvironments can have profound effects on the gas exchange and the uptake and release of micro- and macronutrients through a close association of the autotrophic and heterotrophic organisms and their biological activities [32]. Such patches and different microenvironments may promote the development of diverse communities that are part of food webs. One of the most important groups of microorganisms in the pelagic food web are ciliates [18]. They transfer energy from decaying cyanobacterial blooms to zooplankton via microbial food webs [33].

Ciliates may benefit from diazotrophic cyanobacteria in several ways: (1) by promoting the productivity of ciliate food, meaning the bacteria are supported by the release of organic carbon from decaying cyanobacterial filaments [13], (2) by affecting the metazooplankton community by either harming or favoring taxa, reducing the metazooplankton diversity and the average size, which is important for the development of ciliate (e.g., [10,19]) or (3) by supporting the development of bacteria with nitrogen being released from the cyanobacteria [34]. However, the number of bacteria was not examined in this study, nor was the effect of a ‘low’ or ‘high’ biomass on the physical and chemical conditions of each experiment, so the results should be taken with caution.

Our study showed that different ciliate species, that formed a community, responded differently to diazotrophic cyanobacterium *Aphanizomenon flos-aquae*’s total biomass and morphological form. The ‘high’ biomass of *A. flos-aquae* promoted the development of *Spirostomum* minus and *Euplotes aediculatus*, which was consistent with other experiments, showing that the abundance of bacteria and ciliates was significantly higher after the rapid decay of *A. flos-aquae* [21]. The decaying biomass of *A. flos-aquae* promotes the development of bacteria, that are a favorable food for ciliates. However, both ciliate species preferred the form of individual filaments over the aggregates. *Spirostomum minus* developed quite well on the ‘high’ biomass of the *A. flos-aquae* aggregates, but *Euplotes aediculatus* did not. The poorer development of the ciliates on the biomass of the *A. flos-aquae* aggregates could be explained by the anoxic conditions following the biomass’ decay. Such conditions favor species that are microaerophiles, such as *Spirostomum* [35]. According to Fenchel et al. [36], ciliates survive under anaerobic conditions, but the division rate declines and they are not able to sustain a constant rate of division. The study conducted by Ploug [32] in the Baltic Sea showed that decaying cyanobacterial aggregates provided an anoxic microhabitat (anoxic interior) for about 12 h. These anoxic conditions may favor the development of various bacterial species that do not provide good (or sufficient) food for ciliates. *Spirostomum minus*, the largest species, may also be the most competitive, so it consumes bacteria developing on the aggregates of the *A. flos-aquae* more effectively than the other species.

The ‘low’ biomass filaments and aggregates were the worst conditions for *Strobilidium* sp. This proves that the low biomass of *A. flos-aquae* is a good basis for the development of development, which is advantageous for only some ciliate species. However, single filaments of *A. flos-aquae* were much more preferred than aggregates.

*Vorticella* sp. developed well in the ‘low’ biomass of the aggregates. Previous studies showed that *Vorticella* sp. attached to colonies of cyanobacteria [37], *Anabaena flos-aquae* [38] and *Anabaena lemmermannii* [39]. *Vorticella* stalks adhered directly to the *A. lemmermanni* cells and penetrated the mucus layers. This could indicate that *Vorticella* sp. obtains some nutrients (e.g., nitrogen) directly from the cell of the cyanobacterium. However, it could also be a reciprocal relationship, as *Anabaena* sp. could also benefit from the presence of *Vorticella* sp. The feeding currents that bring particulate morsels to the vorticellids also irrigate the cyanobacteria with solutions of dissolved nutrients [40]. A protozoan respiration could also lead to a local enrichment of CO_2_, which could help alleviate the limitation in a soft water environment. In large numbers, they can form a physical barrier around the cyanobacterium and prevent the predation of its cells by other ciliates that graze the cyanobacterium [38]. The attachment of *Vorticella* sp. to the cyanobacteria colony is also due to its ability of floating that allow for feeding from more areas of the water body and for avoiding predation.

*Paramecium tetraurelia* did not show statistically significant relationships with *A. flos-aquae*, either in single filaments or aggregates, in either a ‘low’ or ‘high’ biomass. There is also a lack of information in the literature. It could be because this species was not found in the habitats with a high development of cyanobacteria.

## 7. Conclusions

Our experiment demonstrated the variability of interactions between the diazotrophic cyanobacterium *Aphanizomenon flos-aquae* and ciliates, but also the variability of benefits that *A. flos-aquae* provides to ciliates. Among them, we identified five potential benefits that we recommend for the subject of a further study:

1. A substrate for the developing of bacteria that serves as food for ciliates.
2. A direct source of nitrogen, carbon and other substances for bacteria that serves as food for ciliates.
3. An indirect source of nitrogen, carbon and other substances for ciliates.
4. An attachment surface for embedded ciliates.
5. A direct source of nitrogen, carbon and other substances for ciliates (*Vorticella* sp.).

## Figures and Tables

**Table 1:** Effect of presence of *A. flos-aquae* total biomass and *A. flos-aquae* single filaments or aggregates on density of particular species of ciliates, Pr—statistical significances are emboldened.

Species of Ciliates		Df	Deviance	Resid. Df	Resid. Dev	Pr (>Chi)
*Spirostomum minus*	NULL (control probe)			44	7063.4	
	CYANO_BIOMASS	2	1632.46	42	5431.0	**<0.001**
	CYANO_MORPHOL	1	279.64	41	5151.3	**<0.001**
*Euplotes aediculatus*	NULL (control probe)			44	103.128	
	CYANO_BIOMASS	2	12.32	42	1112.8	**0.002**
	CYANO_MORPHOL	1	27.71	41	1085.1	**<0.001**
*Strobilidium* sp.	NULL (control probe)			44	2807.2	
	CYANO_BIOMASS	2	171.89	42	2635.3	**<0.001**
	CYANO_MORPHOL	1	241.21	41	2394.1	**<0.001**
*Vorticella* sp.	NULL (control probe)			44	244.06	
	CYANO_BIOMASS	2	12.253	42	231.81	**0.002**
	CYANO_MORPHOL	1	2.331	41	229.48	0.127
*Paramecium tetraurelia*	NULL (control probe)			44	71.818	
	CYANO_BIOMASS	2	2.070	42	69.749	0.3553
	CYANO_MORPHOL	1	0.485	41	69.263	0.4861

**Table 2:** The effect of ‘high’ or ‘low’ biomass of *A. flos-aquae* on the density of ciliate species, *p*—statistical significances are emboldened.

Species of Ciliates	Treatment	Estimate	Std Error	*t* Value	*p* (>|t|)
*Spirostomum minus*	(Intercept) Control (no biomass)	3.353	0.084	40.101	**<0.001**
	‘HIGH’ BIOMASS of cyanobacteria	1.698	0.086	19.860	**<0.001**
	‘LOW’ BIOMASS of cyanobacteria	0.454	0.090	5.048	**<0.001**
*Euplotes aediculatus*	(Intercept) Control (no biomass)	3.509	0.077	45.341	**<0.001**
	‘HIGH’ BIOMASS of cyanobacteria	0.165	0.085	1.939	**0.053**
	‘LOW’ BIOMASS of cyanobacteria	−0.012	0.087	−0.139	0.889
*Strobilidium* sp.	(Intercept) Control (no biomass)	4.489	0.047	94.688	**<0.001**
	‘HIGH’ BIOMASS of cyanobacteria	−0.059	0.053	−1.107	0.268
	‘LOW’ BIOMASS of cyanobacteria	−0.059	0.057	−9.347	**<0.001**
*Vorticella* sp.	(Intercept) Control (no biomass)	0.693	0.316	2.192	**<0.001**
	‘HIGH’ BIOMASS of cyanobacteria	0.574	0.338	1.699	0.089
	‘LOW’ BIOMASS of cyanobacteria	0.926	0.332	2.794	**0.005**
*Paramecium tetraurelia*	(Intercept) Control (no biomass)	1.099	0.258	4.255	**<0.001**
	‘HIGH’ BIOMASS of cyanobacteria	−0.034	0.290	−0.117	0.907
	‘LOW’ BIOMASS of cyanobacteria	0.210	0.283	0.741	0.459

**Table 3:** The effect of *A. flos-aquae* filaments morphology (single filaments or aggregates) on ciliate species, *p*—statistical significances are emboldened.

Species of Ciliates	Treatment	Estimate	Std Error	*t* Value	*p* (>|t|)
*Spirostomum minus*	(Intercept) Control (no cells)	3.353	0.084	40.10	**<0.001**
	AGGREGATES	0.955	0.088	10.90	**<0.001**
	FILAMENTS	1.491	0.086	17.35	**<0.001**
*Euplotes aediculatus*	(Intercept) Control (no cells)	3.509	0.077	45.341	**<0.001**
	AGGREGATES	−0.068	0.087	−0.782	0.434
	FILAMENTS	0.210	0.085	2.473	**0.013**
*Strobilidium* sp.	(Intercept) Control (no cells)	4.489	0.047	94.688	**<0.001**
	AGGREGATES	−0.616	0.057	−10.75	**<0.001**
	FILAMENTS	−0.008	0.053	−0.149	0.882
*Vorticella* sp.	(Intercept) Control (no cells)	0.693	0.316	2.192	**0.028**
	AGGREGATES	0.876	0.332	2.635	**0.008**
	FILAMENTS	0.642	0.336	1.908	0.056
*Paramecium tetraurelia*	(Intercept) Control (no cells)	1.099	0.258	4.255	**<0.001**
	AGGREGATES	0.033	0.288	0.114	0.909
	FILAMENTS	0.154	0.285	0.542	0.588

## Data Availability

Data are available from the first author after request.

## Supplementary Materials

The following supporting information can be downloaded at: https://www.mdpi.com/article/10.3390/ijerph192215097/s1, Table S1. Density of ciliates [ind./mL].
