# Supplementary material for: Interactions between Ciliate Species and Aphanizomenon flos-aquae Vary Depending on the Morphological Form and Biomass of the Diazotrophic Cyanobacterium
Source: Int J Environ Res Public Health. 2022 Nov 16;19(22):15097. doi: 10.3390/ijerph192215097 (PMC9690129; doi:10.3390/ijerph192215097)
Supplement: Supplementary file 1 [file ijerph-19-15097-s001.zip › ijerph-1976628-supplementary.pdf]

Table S1. Density of ciliates [ind./ml]

Day of experiment	BIOMASS of cyanobacteria	Single/aggregated filaments of cyanobacteria	*Spirostomum minus* [ind./1ml]	*Euplotes aediculatus* [ind./1ml]	*Strobilidium* sp. [ind./1ml]	*Vorticella* sp. [ind./1ml]	*Paramecium tetraurelia* [ind./1ml]
0	control sample	control sample	17	85	20	1	3
3	control sample	control sample	15	7	24	1	1
7	control sample	control sample	20	17	105	1	2
10	control sample	control sample	34	18	194	1	2
13	control sample	control sample	57	40	102	6	7
0	high	single filaments	17	85	20	1	3
3	high	single filaments	25	15	21	1.5	1.5
7	high	single filaments	98	20	115	3	1
10	high	single filaments	232	23	192	2	1.5
13	high	single filaments	610	90	240	2.5	6
0	low	single filaments	17	85	20	1	3
3	low	single filaments	31.5	27	34	3.5	4.5
7	low	single filaments	42	17	44.5	1.5	1.5
10	low	single filaments	41.5	8	55	2.5	7.5
13	low	single filaments	155.5	42	140.5	19.5	5.5
0	high	aggregated filaments	17	85	20	1	3
3	high	aggregated filaments	21	10	19.5	2	3
7	high	aggregated filaments	75.5	13.5	51.5	3	2
10	high	aggregated filaments	141.5	15.5	87	2.5	4
13	high	aggregated filaments	325	37	72	17	4
0	low	aggregated filaments	17	85	20	1	3
3	low	aggregated filaments	9.5	10	42	2.5	3.5
7	low	aggregated filaments	33.5	14	63,5	2	1
10	low	aggregated filaments	33.5	8	75.5	4	1

13	low	aggregated filaments	69.5	34	39.5	13	7
